# The Outcomes of Salvage Surgery for Vascular Injury in The Extremities: A Special Consideration For Delayed Revascularization

**DOI:** 10.5704/MOJ.1403.012

**Published:** 2014-03

**Authors:** Krishnan Jagdish, M Paiman, AS Nawfar, MI Yusof, W Zulmi, WS Azman, AS Halim, AZ Mat Saad, MD Shafei, WI Faisham,

**Affiliations:** Department of Orthopaedics, School of Medical Sciences, Universiti Sains Malaysia, Kubang Kerian, Malaysia; Department of Orthopaedics, School of Medical Sciences, Universiti Sains Malaysia, Kubang Kerian, Malaysia; Department of Orthopaedics, School of Medical Sciences, Universiti Sains Malaysia, Kubang Kerian, Malaysia; Department of Orthopaedics, School of Medical Sciences, Universiti Sains Malaysia, Kubang Kerian, Malaysia; Department of Orthopaedics, School of Medical Sciences, Universiti Sains Malaysia, Kubang Kerian, Malaysia; Reconstructive Sciences Unit, School of Medical Sciences, Universiti Sains Malaysia, Kubang Kerian, Malaysia; Reconstructive Sciences Unit, School of Medical Sciences, Universiti Sains Malaysia, Kubang Kerian, Malaysia; Reconstructive Sciences Unit, School of Medical Sciences, Universiti Sains Malaysia, Kubang Kerian, Malaysia; Department of Radiology, School of Medical Sciences, Universiti Sains Malaysia, Kubang Kerian, Malaysia; Department of Orthopaedics, School of Medical Sciences, Universiti Sains Malaysia, Kubang Kerian, Malaysia

## Abstract

A seven years retrospective study was performed in 45 consecutive vascular injuries in the extremities to investigate the pattern of injuries, managements and outcomes.

Motor-vehicle accidents were the leading cause of injuries (80%), followed by industrial injuries (11.1%) and iatrogenic injuries (4.4%). Popliteal and brachial artery injuries were commonly involved (20%). Fifteen (33.3%) patients had fractures, dislocation or fracture dislocation around the knee joint and 6 (13.3%) patients had soft tissue injuries without fracture. Traumatic arterial transection accounted for 34 (75.6%) cases, followed by laceration in 7 (15.6%) and 9 (6.7%) contusions. Associated nerve injuries were seen in 8 (17.8 %) patients using intra-operative findings as the gold standard, both conventional angiogram (CA) and computerized tomography angiogram (CTA) had 100% specificity and 100% sensitivity in determining the site of arterial injuries.

The mean ischemic time was 25.31 hours (4 - 278 hours). Thirty-three (73.3 %) patients were treated more than 6 hours after injury and 6 patients underwent revascularization after 24 hours; all had good collateral circulation without distal pulses or evidence of ischemic neurological deficit. The mean ischemic time in 39 patients who underwent revascularization within 24 hours was 13.2 hours. Delayed amputation was performed in 5 patients (11.1%). Of the 6 patients who underwent delayed revascularization, one patient had early amputation, one -had delayed amputation following infection and multiple flap procedures while the rest of the patients’ limbs survived. Joint stiffness was noted in 10 patients (22.2%) involving the knee joint, elbow and shoulder in two patients each. Infection was also noted in 5 patients (11.1%) with two of them were due to infected implants. Other complications encountered included nonunion (2 patients, 4.4%), delayed union (1 patient, 2.2%),limb length discrepancy (1 patient, 2.2%), hematoma (1 patient, 2.2%) and leaking anastomosis in one patient (2.2%). Volkmann’s ischemic contracture occurred in 3 (6.7%) patients. There was no complication noted in 8 (17.8%) patients Three patients (6.7%) died of whom two were not due to vascular causes. We conclude that early detection and revascularization of traumatic vascular injuries is important but delayed revascularization also produced acceptable results.

## Introduction

Extremities vascular injury has been on the increase due to high velocity injuries following motor vehicles accidents. The definitive management of this problem is often not optimum due to late diagnosis, delayed referrals and lack of expertise in vascular injury management. Local studies revealed that the most common cause of the injury was road traffic accidents. Primary arterial repair was the most common surgical procedure carried out (64%) followed by autologous saphenous vein graft (17%). Amputation, either primary or delayed, was required in 15% of patients and presence of nerve and venous injury led to poor outcomes^1^.

The natural history of untreated extremity vascular injury is not well understood. Late amputation is required due to chronic ischemia, chronic ulceration, arterio-venous fistula and permanent neurological deficits^2^. In our country, most of the patients present later than 6 hours of warm ischemia period due to various reasons. Generally delayed revascularisation is associated with higher amputation rate. However, current literatures show that delayed intervention also produced acceptable outcomes^3,4^. This study was to evaluate the outcomes in our patients who were managed with delayed revascularisation following vascular injuries of the extremities.

## Materials and Methods

### 

A seven-year retrospective study was performed in 45 consecutive vascular injuries in the extremities to investigate the pattern of injuries, treatment and the outcomes in our center from February 2006 till February 2013.

The diagnosis of extremity vascular injuries was made based on clinical evaluation; with absence of distal pulses, pallor, cold distal extremities and confirmed by Doppler ultrasound. The patients with gangrenous extremities evidenced by the presence of mottling were excluded from the study. Preoperative radiological investigations were performed in 35 cases using conventional angiogram (17 cases) and computed tomography scan angiography (CTA) (20 cases). The remaining patients were operated without radiological evaluation, including those with open fractures with large wounds. The parameters analyzed in this study were the timing and findings of CTA and conventional angiography, length of injury-time, type of surgery performed, intraoperative findings (type and length of vascular injury), type of graft and length of graft used, associated fractures and the complications.

The final diagnosis was confirmed intra-operatively with the presence of arterial transection or thrombosis with absence of distal flow upon exploration and intra-operative Doppler confirmation. The viability of the muscles was evaluated by the presence of contraction upon stimulation. Those cases with established non-viable muscles were excluded in this study. After revascularization, the presence of distal perfusion was assessed by Doppler ultrasound and monitored closely for 48 hours. Heparin infusion (10,000 iu) and Dextran 40 were given during the first 24 hours followed by subcutaneous low molecular weight heparin for a week. The patients were discharged with aspirin (150mg daily) for 6 weeks.

## Results

There were 45 patients - in this study. The male to female ratio was 9:1 with the mean age of 25.4 years. The incidence of arterial injury involving the lower limbs (29 patients, 64.4 %) was two times more than the upper limbs (15 patients, 33.3%). One patient had both upper and lower limbs injuries. Vascular injury involving the right extremity was seen in 28 (62.2%) patients and 17 (38.8%) -in the left extremity.

Arterial injury caused by blunt trauma was in 38 (84.4%) patients and 5 (11.1%) due to penetrating injury. The blunt injuries were mostly seen in motor vehicle accidents (80%). Penetrating injuries were seen in 5 (11.1%) patients. Two (4.4%) patients had vascular injury during delayed stabilization of intercondylar fracture and in periprosthetic fracture. (Table I)

Vascular injuries were contributed by femur fractures (22.2%), proximal tibia fractures (20%) and humeral fractures (20%). Fifteen (33.3%) patients had fractures, dislocation or fracture dislocation around the knee joint and 6 (13.3%) patients had soft tissue injuries without fractures. Twenty-six (57.8%) patients had open fractures and 13 (28.9%) had closed fractures. Popliteal artery injuries were in 33.3% and brachial artery in 20% of cases. In 34 (75.6%) of cases, the arterial injuries were of traumatic transection compared to 7 (15.6%) laceration and 9 (6.7%) contusions. One patient developed pseudo aneurysm.(Table I)

**Surgical intervention** Thirty-nine patients had acute limb ischemia following injuries and were revascularized within 24 hours. The mean ischemic time of these patients was 13.2 hours. Forty patients underwent reverse saphenous vein graft reconstruction and 5 - had polytetrafluoroethylene (PTFE) synthetic graft bypass. The mean length of arterial injury was 8.5 cm (3.0 - 21.0 cm)

The mean ischemic time was 25.3 +/- 4.3 hours ( 4 - 278 hours). Most of the patients, totalling 33 (73.3 %) were revascularised after 6 hours. Six patients underwent revascularization after 24 hours and they had good collateral circulation without distal pulses and evidence of ischemic neurological deficit. The mean duration of revascularization in these patients was 103 hours (4.3 days). (Table I)

**Outcomes** Eighty-nine percent of the limbs survived after surgical intervention. In 6 patients with delayed revascularization, one had early amputation and one delayed amputation due to infection. The rest of the patients’ limbs survived.

Delayed amputation was performed in 5 (11.1%) patients All of them had high velocity blunt injuries with fractures of the femur or proximal tibia or both. The ischemic time was 4, 12, 15, 84 and 278 hours respectively. Joint stiffness was noted in 10 patients (22.2%); the knee in 6 patients, elbow in 2 and shoulder in 2 patients Infection was also noted in 5 patients (11.1%), of which 2 were due to infected implants. Other complications encountered were non-union 2 (4.4%), delayed union 1 (2.2%), limb length discrepancy 1 (2.2%), hematomas 1 (2.2%) and leaking anastomosis 1 (2.2%). Volkmann ischemic contracture was seen in 3 patients (6.7%).

Three (6.7%) patients died, two due to non-vascular causes and one due to thrombosed external iliac vessels with multiple pelvic fractures. He died after 10 days due acute renal failure and delayed sepsis.

Eight (17.8 %) patients had associated nerve injuries. Three (6.7%) patients had brachial plexus injury, one (2.2%) had ulnar nerve palsy and 4 patients (8.9%) had common peroneal nerve injury. Two patients with peroneal nerve injury had motor recovery with persistent numbness over the dorsum of the foot. Two patients had foot drop at the time of review.

The intraoperative findings correlated 100% with preoperative CT angiogram and conventional angiogram with regards to presence and site of vascular injuries.

(Table Ia)

(Case 1)

(Case 2)

(Case 3)

## Discussion

In Malaysia, vascular injuries associated with fractures of the extremities are increasing yearly. Motor vehicle accidents (MVA) were the leading cause of injuries making up 80% of cases, followed by 11.1% industrial injury and 4.4% iatrogenic injury. Motorcycle accidents contributed to 95.6% of cases. There were 84.4% closed blunt injuries and 11.6% penetrating injuries

The vast majority of the patients (91.1%) were male (n =41) with mean age of 25.4 (7-66 years). This finding is similar to other studies^5,6,7,8^. A local study in 2002, reported that male patients consisted of more than 90% of the patients and involved, mainly young adults in the range of 21 to 30 years of age1. We noted that 36 or 80% of the patients sustained vascular injuries due to MVA, - 94.4% of them were motorcyclist. Motorcyclists are more susceptible because of the high-energy trauma to the unprotected extremities. The effect of high-energy trauma was clearly recognized as 83.3% of the motorcyclist involved had a collision with heavy vehicles.

The most common artery injured was the popliteal artery (33.3%) followed by femoral artery (24.4%). These findings were consistent with other studies^1,9,10^. In our study, the incidence of popliteal artery injury was predominant in patients with fractures and/or dislocations around the knee joint. The incidence of popliteal artery injury in association with knee dislocation can be as high as 32%^5,7,8^. The popliteal artery is very liable to injury due to its anatomical course, whereby it is anchored above and below the knee joint as it passes through the popliteal fossa^9^. In the upper limb, the brachial artery was the most commonly injured artery (20%). All the brachial artery injuries were caused by blunt trauma secondary to humeral fractures.

Conventional angiography (CA) was performed on 17 patients with suspected vascular injury with extremity trauma, whereas- 20 patients underwent CTA. There was 100% specificity and 100% sensitivity of both CA and CTA with regards to the location of arterial injury which were correlated with intra-operative findings. Both CA and CTA were effective to diagnose vascular injuries. The diagnostic accuracy of CTA in our study was similar to other prospective studies^10,11^. We recommended radiological evaluation either by CTA or angiogram as a standard procedure prior to surgical intervention.

The nature of the arterial injury included transection (either total or partial), laceration, contusion or aneurysmal sac. Transection was the most common form of arterial injury comprising 34 (75.6%) patients. Due to insufficient documentation in the operative notes, we were unable to determine the exact number of total or partial injuries. On the other hand, laceration type of injury was reported in 7 (15.6%) patients and out of this 6 (85.71%) occurred in the upper limb. A prospective study involving 550 patients, reported that transection type was not only the most common type, but also associated with the highest risk of limb loss^12^. Vascular injuries were most commonly caused by femur fractures (22.2%), followed by proximal tibia and humeral fractures (20%). This implies that vascular injuries are associated with high-energy trauma, especially in blunt injuries. Femoral fractures were associated with injuries to the femoral artery, profunda femoris artery, superficial femoral and popliteal arteries. Twenty-six (57.8%) patients had open fractures and 13 (28.9%) patients closed fractures. Incidence of arterial injury in the lower limbs was almost double- compared to upper limbs (64.4% versus 33.3%). This finding was similar to other studies^1,5,6,10,12^.

The mean ischemic time in our study was 25.3 hours which was beyond the “golden period” of 6 hours warm ischemic time, proposed by many authors to maximize limb salvage^13,14^. The ischemic time in our study ranged from 4 to 278 hours. Thirty-three (73.3 %) patients were treated more than 6 hours after injury. Thirty-nine patients were considered to have acute limb ischemia following injuries and were revascularized within 24 hours. The mean ischemic time of these patients was 13.2 hours. The overall extremities salvage rate in our patients was 88.9 % (40/45). The amputation rate reported varied between 4.8% and 86%^14,15^. This is considerably low given the fact that most of the revascularization were done after lapse of the golden period. Furthermore, Volkmann ischemic contracture secondary to prolonged ischemia was noted in only 3 (6.7%) patients. Functional outcome was influenced more by the nature and pattern of injury rather than the ischemic time. As such, limb viability cannot be predicted by ischemic time alone.

Huynh et al concluded that even though prolonged ischemia has been a well-recognized predictor of cell death, the tolerance period varies from person to person. This depends on the severity of the ischemia and the presence of collateral flow^16^. Another study concluded that the greatest impact on decision making between limb salvage and amputation depended on the severity of soft tissue injury^17^. There were three mortalities (6.7%) in our study. One patient had external iliac thrombosis with multiple pelvic fractures. Complex pelvic injury with vascular injury is associated with high mortality despite aggressive and immediate treatment^18^.

Six patients underwent revascularization after 24 hours and they had good collateral circulation without distal pulses or evidence of ischemic neurological deficits. The mean duration of revascularization in these patients was 103 hours (4.3 days). Delayed revascularization after popliteal trauma in selected cases could improve motor functions. Gastrocnemius muscle viability, intact distal sensation without skin mottling had been shown to be good predictor in these cases. Presence of collateral circulation- would enable foot viability, but not to maintain motor function in majority of cases due to muscle ischemia. The late revascularization with by-pass graft of injured vessels will improve limb perfusion and reduce muscle ischemia^3,4^. Furthermore, delayed repair of brachial artery injury also produced similar result. The long-term functional outcome of the injured limb was also dependent on recognition and successful treatment of associated nerve injuries. Therefore, time should not be the only factor for consideration of revascularization in vascular injuries.

Open surgery with primary repair, vein patch, and interposition or bypass grafting had been the gold standard for management of vascular injuries^19^. Forty (88.9%) patients underwent interposition with contralateral reverse saphenous vein graft, while 5 patients used PTFE (11.1%). PTFE was generally used to replace large caliber vessels such as femoral artery. Autologous saphenous vein grafting has stood the test of time. It has better patency rates and resistance to infection compared with synthetic grafts^19^.

**Table I: Detailed description of vascular injuries:**
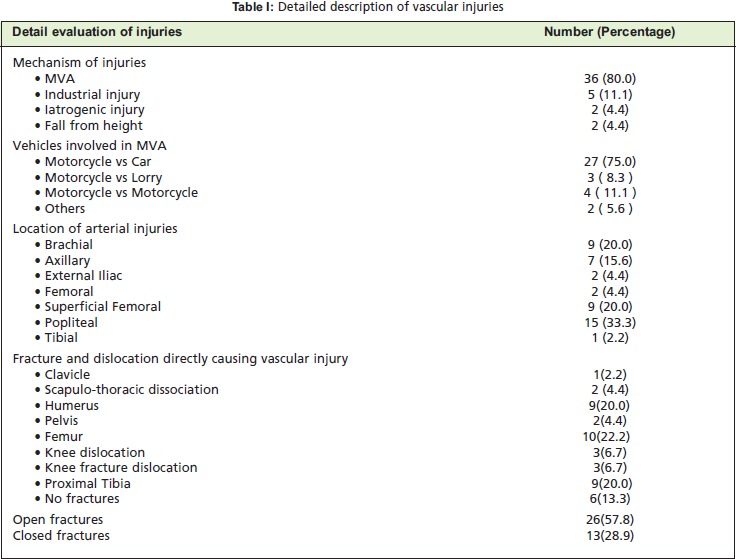


**Cases of delayed revascularization:**
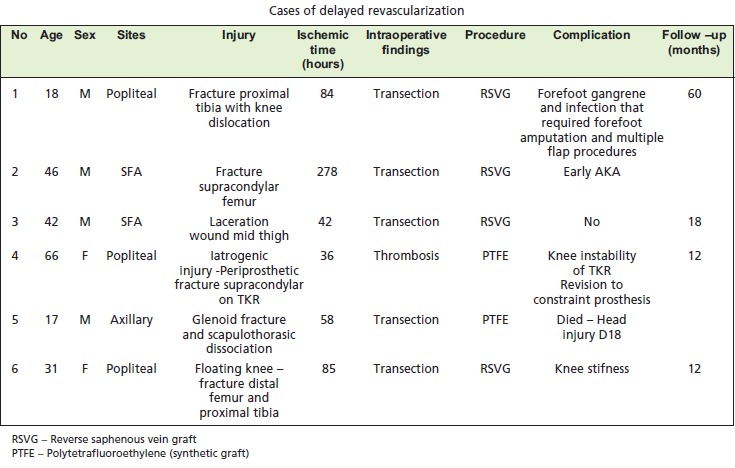


**Case 1: 23- year old motorcyclist collided with car and sustained closed knee dislocation. Immediate reduction did not return distal pulses. The CTA showed complete block of popliteal artery with good distal run-off. Intra-operatively thrombosis of vessels confirmed with tears of posterior capsule and ligament.**
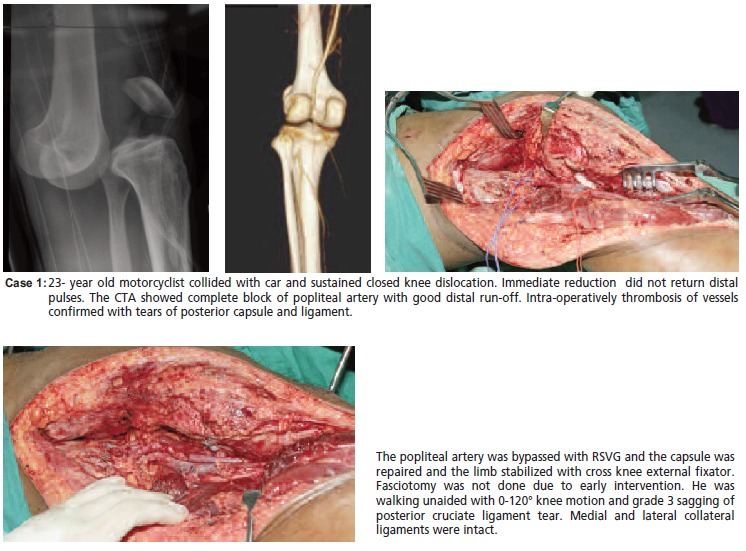


**Case 2: 36-year old motorcyclist collided with car and sustained scapula-thoracic injury associated with -proximal humerus fracture. He had complete loss of upper limb motor function and absence of both distal pulses.**
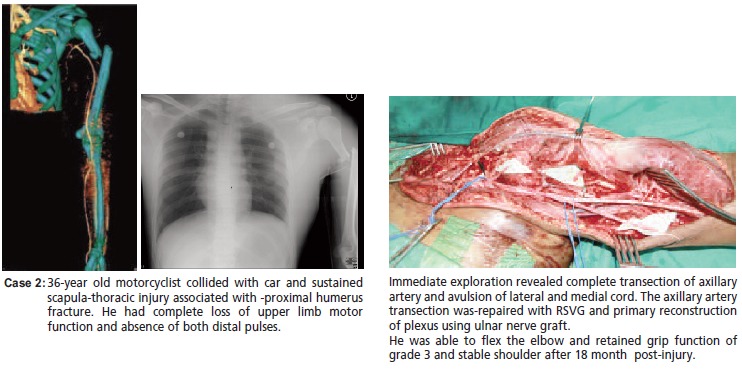


**Case 3: 20-year old motorcyclist collided with lorry and sustained bilateral femur fracture. He presented with compartment syndrome of the thigh and immediate fasciotomy was done but distal pulses not detected.**
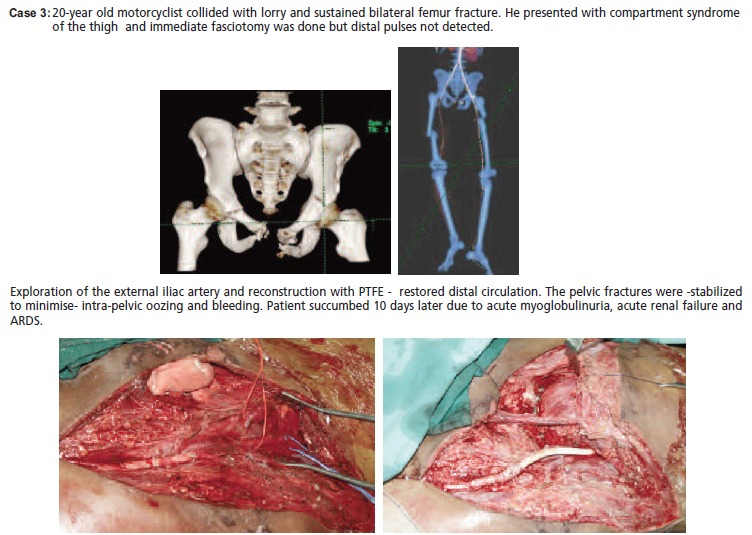


## Conclusion

Early detection and revascularization of traumatic vascular injuries is important but delayed revascularization also produces acceptable salvage outcomes.
